# Comparison of Stanford and DeBakey Classification Distributions Between Hospital-Diagnosed and Forensic Autopsy-Diagnosed Aortic Dissection Cases

**DOI:** 10.3390/jcdd13090417

**Published:** 2026-08-26

**Authors:** Mucahit Oruç, İsmail Altın, İlker Gazioğlu, Uğur Demir, Ömer Turan

**Affiliations:** 1 Department of Forensic Medicine, Faculty of Medicine, İnönü University, 44280 Malatya, Türkiye; mucodr@gmail.com; 2 Department of Forensic Medicine, Faculty of Medicine, Istanbul Medeniyet University, 34722 Istanbul, Türkiye; omerturan@gmail.com; 3 Council of Forensic Medicine, Şanlıurfa Branch Office, Ministry of Justice, 63100 Şanlıurfa, Türkiye; ilker_gazioglu12@hotmail.com; 4 Department of Forensic Medicine, Faculty of Medicine, Harran University, 63300 Şanlıurfa, Türkiye; ugurdmr81@gmail.com

**Keywords:** aortic dissection, autopsy, forensic medicine, Stanford classification, DeBakey classification

## Abstract

**Background**: This study compared demographic characteristics and Stanford and DeBakey classification distributions between aortic dissection cases diagnosed in a tertiary hospital and those diagnosed at forensic autopsy. **Methods**: This retrospective comparative study included 58 cases diagnosed between 31 December 2019, and 1 January 2024. Hospital-diagnosed cases (*n* = 31) were identified from electronic medical records, whereas cases diagnosed at autopsy (*n* = 27) were retrieved from medico-legal autopsy reports. Demographic characteristics and classification distributions were compared. **Results**: Mean age was higher among hospital-diagnosed cases than among forensic autopsy-diagnosed cases (52.84 ± 17.59 vs. 43.93 ± 17.26 years; mean difference: 8.91 years; 95% CI: −0.27 to 18.10; *p* = 0.057). Stanford type A was more frequent among forensic autopsy-diagnosed cases (92.6% vs. 67.7%), with an absolute difference of 24.9 percentage points (95% CI: 3.8 to 43.2; Fisher’s exact *p* = 0.025). The overall DeBakey distribution also differed between the groups (Fisher–Freeman–Halton exact *p* = 0.044), with type III being 24.9 percentage points less frequent among forensic autopsy-diagnosed cases (95% CI: 3.8 to 43.2). Sex was not significantly associated with either classification. **Conclusions**: Stanford type A and DeBakey types I–II predominated among cases diagnosed at autopsy. These findings demonstrate classification differences according to diagnostic setting and emphasize the contribution of forensic autopsy to identifying fatal aortic dissections.

## 1. Introduction

Cardiovascular diseases are estimated to account for approximately 17 million deaths annually worldwide [1,2]. In the United States (USA), 36% of deaths have been reported to be of cardiovascular system (CVS) origin [3]. According to the Turkish Statistical Institute (TÜİK), 36.8% of all deaths in Türkiye are due to circulatory system diseases [4].

The aorta, known as the largest artery in the body, carries oxygenated blood from the heart to other regions. The aorta is the largest elastic artery in the human body and is classically divided into thoracic and abdominal segments. The thoracic aorta comprises the aortic root, ascending aorta, aortic arch, and descending thoracic aorta [5]. Aortic dissection refers to blood entering through a tear in the aortic intima and tracking within the medial layer, resulting in separation of the aortic wall and formation of a true and a false lumen. As blood continues to flow into the aortic wall, the intimal flap may propagate antegrade and retrograde from the initial tear or hemorrhage site, and may even extend into collateral arteries. Aortic dissection is a life-threatening condition characterized by sudden chest and/or back pain [6,7,8].

Estimating the incidence of aortic dissection is challenging because many cases are only diagnosed postmortem. The incidence is estimated at 2.0–3.5 per 100,000. Studies from Sweden have shown that the incidence of aortic dissection is increasing [9]. Men are more frequently affected: approximately 65% of all patients with aortic dissection are male. The mean age at diagnosis is 63 years [8].

**Figure 1:**
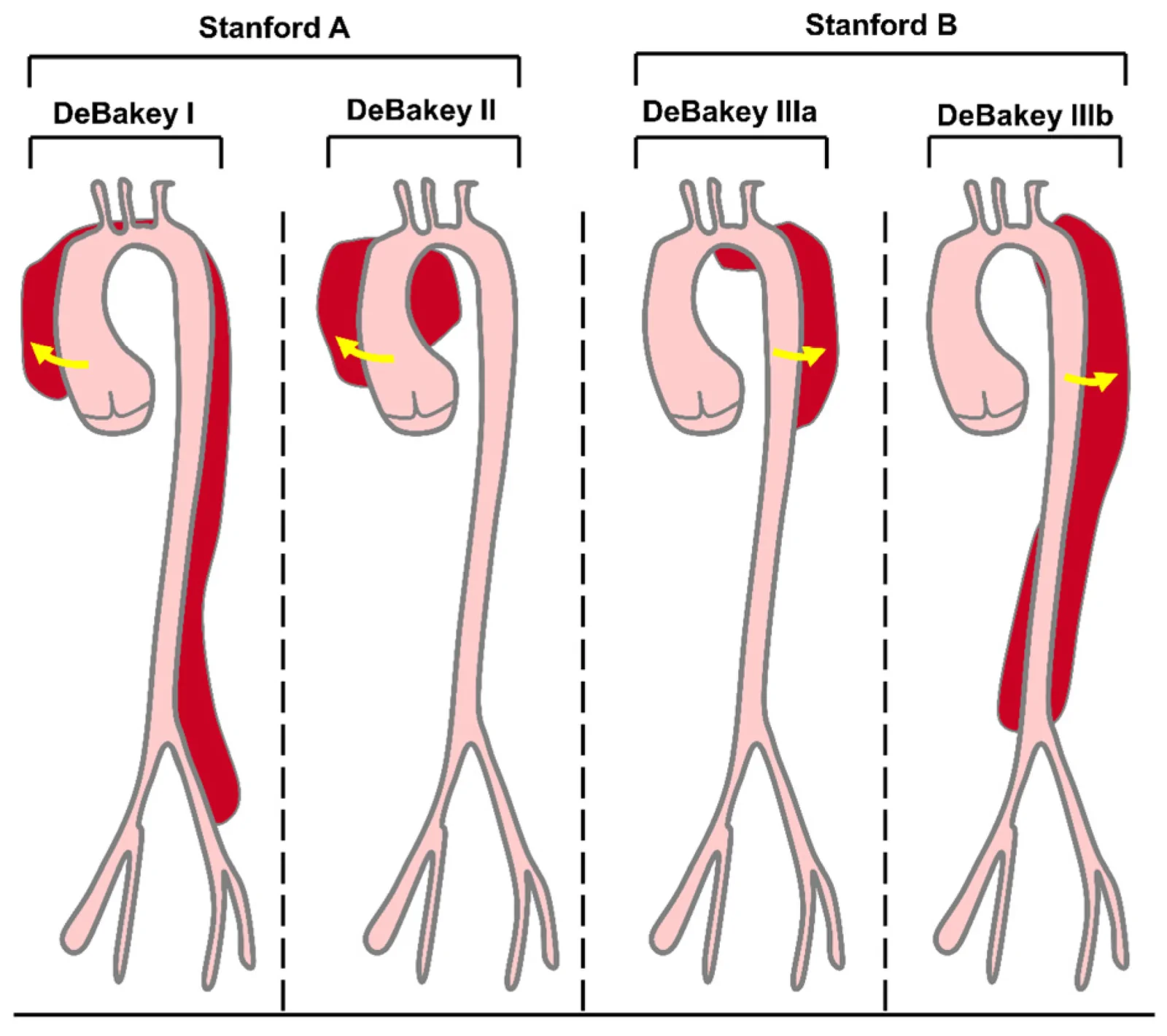
DeBakey and Stanford classifications of aortic dissection (AD). The DeBakey classification is based on the site of origin and anatomical extent of the dissection. DeBakey type I involves the ascending aorta, aortic arch, and descending aorta; type II is confined to the ascending aorta; and type III spares the ascending aorta. The Stanford classification is based on ascending aortic involvement: Stanford type A involves the ascending aorta, whereas Stanford type B does not involve the ascending aorta. Reproduced from Ref. [12] under the terms and conditions of the Creative Commons Attribution (CC BY 4.0) license.

Classification systems for aortic dissections aim both to describe the pathology and to guide clinical decision-making by identifying appropriate treatment strategies [10,11]. The most commonly used systems are the Stanford and DeBakey classifications (Figure 1) [10,11]. DeBakey type I involves the proximal aorta, aortic arch, and descending thoracic aorta; DeBakey type II involves only the ascending aorta; and DeBakey type III originates in the descending aorta distal to the left subclavian artery and may remain confined to the thoracic aorta (type IIIa) or extend below the diaphragm (type IIIb). In contrast, the Stanford system distinguishes dissections by involvement of the ascending aorta: Stanford type A (corresponding to DeBakey types I–II) involves the ascending aorta and almost always necessitates urgent open surgical repair, whereas Stanford type B (corresponding to DeBakey type III) involves the descending aorta and is generally managed with medical therapy and/or thoracic endovascular aortic repair (TEVAR) [10,11,12].

Forensic autopsies play a critical role in identifying cases of aortic dissection that were not diagnosed during life and in documenting their anatomical features, thereby contributing to public health surveillance. However, postmortem diagnosis alone does not establish that a diagnosis was clinically missed or misinterpreted, as such a determination requires detailed information regarding the premortem clinical course and available diagnostic opportunities. Therefore, the combined evaluation of clinical and forensic data may provide a more comprehensive perspective on the recognition and anatomical distribution of fatal aortic dissection [1,13].

Against this background, the present study aimed to compare demographic characteristics and the distributions of Stanford and DeBakey classifications between aortic dissection cases diagnosed in a tertiary university hospital and those first identified during forensic autopsy. By evaluating these two diagnostic settings together, we sought to characterize differences in the anatomical distribution of aortic dissection and to provide an integrated clinical and forensic perspective on its recognition.

## 2. Materials and Methods

Between 31 December 2019, and 1 January 2024, we retrospectively evaluated 58 aortic dissection cases: 31 cases diagnosed in a university hospital in a city in eastern Türkiye and 27 cases diagnosed at autopsy.

### 2.1. Study Design and Setting

This retrospective observational study was conducted to evaluate aortic dissection cases diagnosed clinically in a tertiary university hospital and those identified postmortem at the regional Forensic Medicine Institution between 31 December 2019, and 1 January 2024. The hospital serves as a tertiary referral center with cardiovascular surgery, radiology, and cardiology units capable of advanced imaging and surgical management. The autopsy data were obtained from medico-legal postmortems performed under the authority of the Council of Forensic Medicine.

### 2.2. Study Population and Comparative Framework

The study population comprised two case series defined according to the setting in which the diagnosis of aortic dissection was established. The hospital group included 31 patients diagnosed during clinical evaluation at the tertiary university hospital, whereas the forensic group included 27 deceased individuals in whom the diagnosis was first established during medicolegal autopsy. Because group allocation was determined by the diagnostic setting, the two groups were not matched and were not assumed to represent epidemiologically equivalent source populations. The comparison was designed as a descriptive and exploratory assessment of demographic characteristics and Stanford and DeBakey classification distributions across clinical and forensic settings. Accordingly, the numbers of cases in the two groups should not be interpreted as representing the relative frequencies of clinically diagnosed and postmortem-diagnosed aortic dissection in the general population.

### 2.3. Case Selection

Cases with a confirmed diagnosis of aortic dissection were retrospectively identified from two data sources: the electronic hospital information system and forensic autopsy protocols with accompanying histopathology reports. The inclusion criteria were (i) confirmation of aortic dissection by imaging, surgery, macroscopic autopsy findings, or histopathological examination and (ii) availability of sufficient demographic and diagnostic information for classification. Cases involving traumatic aortic rupture, advanced decomposition that prevented reliable classification, or records lacking sufficient information to confirm and anatomically classify the dissection were excluded. Eligible cases were categorized according to the diagnostic setting as hospital-diagnosed cases and cases in which the diagnosis was first established during forensic autopsy.

#### 2.3.1. Forensic Cohort Identification and Postmortem Classification

During the study period, a total of 5400 medicolegal autopsies were performed at our center. The National Judiciary Informatics System (UYAP) records of these autopsies were electronically screened using the term “aortic dissection” and related keywords. All records retrieved through this search were individually reviewed. Cases were included only when aortic dissection was identified during complete autopsy and confirmed by macroscopic and histopathological examination. All 27 cases meeting these eligibility criteria were included consecutively, and no additional sampling or case selection was performed. These individuals had been referred to our center as suspicious death cases requiring medicolegal investigation and underwent complete autopsy. Aortic dissection was identified by direct macroscopic examination of the aorta during autopsy and was subsequently confirmed by microscopic examination of the collected tissue samples. Macroscopic diagnosis was based on the identification of an intimal tear, separation of the layers of the aortic wall, and formation of true and false lumina, whereas histopathological examination demonstrated disruption of the aortic media and intramural hemorrhage. Stanford and DeBakey classifications were assigned according to established anatomical criteria [10,11]. Stanford classification was based on the presence or absence of ascending aortic involvement, whereas DeBakey classification was determined according to both the site of origin and the anatomical extent of the dissection. When classification was ambiguous, the macroscopic and histopathological findings were reviewed by two forensic pathologists, and the final classification was established by consensus.

#### 2.3.2. Hospital Cohort Identification

The hospital cohort was identified retrospectively by querying the hospital electronic information system using ICD codes corresponding to aortic dissection. All records retrieved through this search were individually reviewed. Inclusion required confirmation of the diagnosis from the available medical records and the availability of sufficient demographic and anatomical information to assign Stanford and DeBakey classifications. The diagnostic assessment was based on the available radiological and/or operative documentation. All cases meeting these eligibility criteria were included, and no additional sampling was performed. Records in which the diagnosis could not be confirmed or the anatomical information was insufficient for classification were excluded. For hospital-diagnosed cases, Stanford and DeBakey classifications were established retrospectively from the anatomical findings documented in the original radiology reports and, when available, operative reports. The original imaging studies were not independently reinterpreted for the present study. Classification was based on the standard anatomical definitions of the Stanford and DeBakey systems [10,11]. Case identification was not restricted according to surgical treatment status or route of hospital admission.

### 2.4. Diagnostic Criteria and Classification

In the hospital group, the diagnosis was established by computed tomography angiography (CTA), echocardiography, or intraoperative observation. In autopsy cases, the diagnosis was confirmed macroscopically by visualizing a tear in the intimal layer with separation of the aortic wall and presence of true and false lumina, supported by histopathological confirmation of medial disruption and hemorrhage. All cases were classified according to both the Stanford and DeBakey systems based on the site of origin and extent of dissection. DeBakey types IIIa and IIIb were analyzed together as DeBakey type III.

### 2.5. Data Collection and Variables

Demographic variables, including age and sex, and aortic dissection classifications, including Stanford type A/B and DeBakey types I–III, were recorded for all eligible cases. All data were anonymized, coded, and prepared for statistical analysis. For the forensic group, premortem clinical information was not systematically available in the medicolegal files. Therefore, prior symptoms, healthcare encounters, presumptive diagnoses, diagnostic investigations, and the interval from presentation to death could not be analyzed. The designation “diagnosed at autopsy” refers solely to the setting in which the definitive diagnosis was established and does not indicate that the diagnosis was clinically missed.

### 2.6. Statistics

Statistical analyses were performed using SPSS for Microsoft Windows, version 21.0 (IBM Corp., Armonk, NY, USA). The distribution of continuous variables was assessed using the Shapiro–Wilk test. Continuous variables were summarized as mean ± standard deviation (SD) and, where appropriate, median and interquartile range (IQR). Categorical variables were presented as counts and percentages. Because the overall age distributions did not show significant deviation from normality, age was compared between the hospital-diagnosed and forensic autopsy-diagnosed groups using Welch’s independent-samples *t*-test. Because of the small sex-stratified subgroup sizes, age comparisons within male and female subgroups were performed using two-sided Mann–Whitney U tests with exact permutation *p*-values. Mean differences were calculated as hospital-diagnosed minus forensic autopsy-diagnosed values, and their 95% confidence intervals were estimated using Welch’s method. Categorical comparisons involving two-category variables were performed using Fisher’s exact test, whereas comparisons involving the three-category DeBakey classification were performed using the Fisher–Freeman–Halton exact test. For the Stanford type A and DeBakey type III comparisons, absolute percentage-point differences and their 95% confidence intervals were calculated using the Newcombe method based on Wilson score intervals. All tests were two-sided, and *p* < 0.05 was considered statistically significant. Given the limited sample size and restricted availability of potential covariates, no multivariable modeling was performed.

## 3. Results

**Figure 2:**
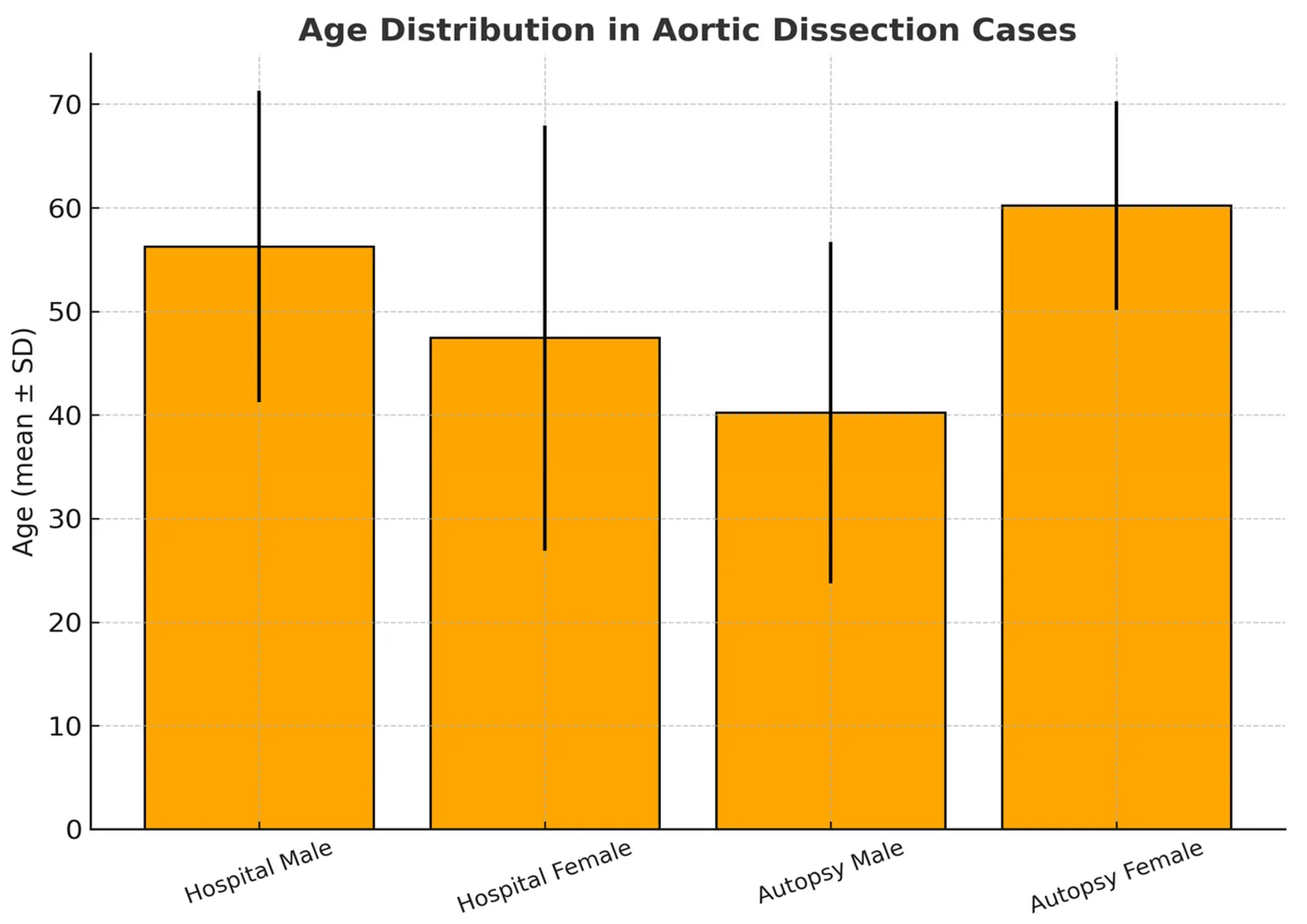
Mean age by sex and diagnostic setting. Error bars represent standard deviations.

During the study period, among 31 aortic dissection cases presenting to the hospital, 19 (61.3%) were male with a mean age of 56.26 ± 15.03 years (range 24–79), and 12 (38.7%) were female with a mean age of 47.42 ± 20.54 years (range 21–81). The mean age for all hospital cases was 52.84 ± 17.59 years (range 21–81). Among 27 aortic dissection cases that underwent autopsy, 22 (81.5%) were male with a mean age of 40.23 ± 16.49 years (range 9–67), and 5 (18.5%) were female with a mean age of 60.20 ± 10.06 years (range 47–75). The mean age for all autopsy cases was 43.93 ± 17.26 years (range 9–75) (Figure 2). Among hospital-diagnosed cases, 8 of 31 (25.8%) were younger than 40 years and 2 (6.5%) were younger than 30 years. Among forensic autopsy-diagnosed cases, 11 of 27 (40.7%) were younger than 40 years, including 7 (25.9%) who were younger than 30 years.

When the groups were compared irrespective of sex, the mean age was 8.91 years higher in the hospital-diagnosed group than in the forensic autopsy-diagnosed group (95% CI: −0.27 to 18.10); however, this difference did not reach statistical significance (Welch’s t(55.19) = 1.94; *p* = 0.057). Among males, the mean ages were 56.26 ± 15.03 and 40.23 ± 16.49 years, respectively, corresponding to a mean difference of 16.04 years (95% CI: 6.07 to 26.00). The respective median ages were 54.0 years (IQR: 46.0–67.5) and 41.0 years (IQR: 25.0–52.5), and the non-parametric comparison was statistically significant (Mann–Whitney U = 98.5; exact *p* = 0.003). Among females, the mean ages were 47.42 ± 20.54 and 60.20 ± 10.06 years, respectively, corresponding to a mean difference of −12.78 years (95% CI: −28.72 to 3.15). The respective median ages were 39.0 years (IQR: 33.0–64.5) and 61.0 years (IQR: 57.0–61.0), and the difference was not statistically significant (Mann–Whitney U = 18.0; exact *p* = 0.225).

Among males, the proportions of DeBakey types I, II, and III were 34.1%, 48.8%, and 17.1%, respectively, compared with 29.4%, 41.2%, and 29.4% among females. The DeBakey distribution did not differ significantly by sex (Fisher–Freeman–Halton exact *p* = 0.609). Stanford type A accounted for 82.9% of male cases and 70.6% of female cases, and the Stanford distribution was also not significantly associated with sex (Fisher’s exact *p* = 0.307) (Table 1). Sex was not significantly associated with either Stanford or DeBakey classification in the overall study group.

Stanford type A was identified in 25 of 27 forensic autopsy-diagnosed cases (92.6%) and 21 of 31 hospital-diagnosed cases (67.7%). Thus, Stanford type A was 24.9 percentage points more frequent among forensic autopsy-diagnosed cases (95% CI: 3.8 to 43.2; Fisher’s exact *p* = 0.025). The overall distribution of DeBakey types also differed between the diagnostic settings (Fisher–Freeman–Halton exact *p* = 0.044). DeBakey type III was identified in 2 of 27 forensic autopsy-diagnosed cases (7.4%) and 10 of 31 hospital-diagnosed cases (32.3%), corresponding to a 24.9-percentage-point lower proportion among forensic autopsy-diagnosed cases (95% CI: 3.8 to 43.2) (Table 2).

## 4. Discussion

Aortic dissection is a rapidly progressive cardiovascular emergency with high mortality that threatens life even when diagnosed. Accordingly, the classifications applied during the diagnostic process and the resulting treatment planning are of critical importance. In this study, we compared the distributions of Stanford and DeBakey classifications between hospital-diagnosed cases and cases diagnosed at autopsy. The findings demonstrated significant differences in classification patterns between the two diagnostic settings. Stanford type A dissections are high-risk conditions that typically require urgent surgery and, if left untreated, may be associated with a mortality rate of up to 50% within the first 48 h [13,14,15]. In the present study, Stanford type A was more frequent among cases diagnosed at autopsy than among hospital-diagnosed cases. Similarly, DeBakey type III was less frequent among cases diagnosed at autopsy than among hospital-diagnosed cases. These findings indicate that proximal dissections were more frequently represented among cases diagnosed at autopsy. Although the absolute differences of 24.9 percentage points indicate a substantial observed contrast between the two diagnostic settings, the relatively wide confidence intervals reflect considerable imprecision arising from the limited sample size. These estimates should therefore be interpreted as exploratory, unadjusted differences between the study groups rather than as precise population-level effects.

The incidence of aortic dissection is known to increase with age, with the risk being particularly elevated in individuals older than 60 years [16,17]. In the present study, the mean age was higher among hospital-diagnosed cases than among cases diagnosed at autopsy; however, the overall difference was not statistically significant. In sex-specific comparisons, hospital-diagnosed male cases were significantly older than male cases diagnosed at autopsy, whereas no significant age difference was observed among female cases. These findings reflect differences in age distribution between the two diagnostic settings and should not be interpreted as evidence of age-related risk or delayed clinical recognition. Moreover, the small number of female cases diagnosed at autopsy limits the interpretation of sex-specific findings. The relatively young age distribution of the forensic cohort is noteworthy in light of the established increase in the incidence of aortic dissection with advancing age [16,17], with 40.7% of cases younger than 40 years and 25.9% younger than 30 years. Hypertension, known aortic aneurysm, bicuspid aortic valve, heritable connective-tissue disorders or genetic aortopathies, family history, and pregnancy or postpartum status are clinically relevant predisposing factors or contexts [15]. However, information on these variables was not consistently available in the forensic records. Therefore, the potential contribution of these conditions to the younger age distribution of the forensic cohort could not be determined. Although the age difference among males remained statistically significant in the non-parametric analysis, all sex-stratified findings should be regarded as exploratory because of the small subgroup sizes, particularly the inclusion of only five female forensic cases, and the resulting imprecision of the estimates.

The identification of aortic dissection for the first time during medicolegal autopsy demonstrates the contribution of postmortem examination to recognizing fatal aortic disease and documenting its anatomical extent. Acute aortic dissection may present with variable clinical manifestations, making prompt recognition and management essential [14,17]. However, cases diagnosed at autopsy should not be interpreted uniformly as missed clinical diagnoses. The available records did not consistently provide information on premortem symptoms, previous healthcare encounters, diagnostic investigations, or the interval from presentation to death; therefore, the reasons why the diagnosis was not established during life could not be determined. In addition, because the hospital and forensic groups were drawn from different diagnostic settings and were not matched source populations, the greater representation of Stanford type A dissection in the forensic group may reflect differences in case selection, disease severity, or pathways leading to medicolegal autopsy. Accordingly, the present findings should be regarded as a descriptive comparison of anatomical distribution across two diagnostic settings rather than as evidence of differential diagnostic performance.

From a contemporary clinical perspective, CT angiography plays a central role in the rapid evaluation of suspected acute aortic syndromes by enabling visualization of the entire aorta, the extent of dissection, branch-vessel involvement, and rupture-related complications [7,15,17]. In patients with atypical or nonspecific presentations, maintaining appropriate clinical suspicion and performing timely CT angiography when acute aortic syndrome is included in the differential diagnosis may help reduce delayed recognition. However, the present study lacked sufficiently detailed premortem clinical information to determine whether CT angiography was indicated, available, or performed in the forensic cases or whether its use would have altered the diagnosis or outcome.

We observed statistically significant differences in the distributions of both Stanford and DeBakey classifications between hospital-diagnosed cases and cases diagnosed at autopsy. The predominance of proximal dissections among cases diagnosed at autopsy highlights the importance of detailed anatomical classification in forensic evaluation. A recent study comparing surgically treated and autopsied acute type A aortic dissection cases similarly emphasized the complementary contribution of clinical and postmortem data [18]. At forensic autopsy, detailed anatomical examination is essential for documenting the origin and extent of the dissection and its fatal complications [19]. Aortic dissection has also been reported as a subject of medical malpractice litigation, particularly in cases involving alleged failure or delay in diagnosis [20]. In such cases, autopsy findings may provide objective medical evidence for the subsequent medicolegal assessment; however, the determination of medical liability requires consideration of the clinical presentation, available diagnostic opportunities, medical records, and applicable standards of care.

Taken together, these findings demonstrate the complementary descriptive value of clinical and forensic datasets while also underscoring the need for caution when interpreting differences between diagnostic settings [18]. The present study did not evaluate presenting symptoms, premortem healthcare encounters, diagnostic pathways, imaging practices, treatment, or clinical outcomes; therefore, it cannot determine whether alternative diagnostic or management strategies would have changed the diagnosis or outcome in individual cases. Population-based research incorporating hospital and forensic autopsy databases has demonstrated the importance of comprehensive case ascertainment in characterizing the full spectrum of acute aortic dissection [21]. Future studies linking detailed premortem clinical records with standardized autopsy findings are needed to investigate the factors associated with aortic dissections first identified after death.

### Study Limitations

This study has several limitations. First, its retrospective design and relatively small sample size, particularly the limited number of female cases diagnosed at autopsy, reduce the statistical power of subgroup analyses. Second, hospital-diagnosed cases and cases diagnosed at autopsy were obtained from different data sources that may not represent fully overlapping catchment populations, potentially introducing selection and referral bias. The groups were not matched, and the analyses were not designed to control for potential differences in case selection, clinical presentation, disease severity, referral pathways, or access to diagnostic evaluation. Consequently, the observed differences represent unadjusted associations and cannot be attributed solely to the diagnostic setting. Third, differences in the content and completeness of hospital records and forensic autopsy reports may have resulted in information bias. Finally, because the study was not population-based, the proportion of cases diagnosed at autopsy and the observed classification distributions should not be interpreted as estimates of incidence, diagnostic failure, or mortality risk. These limitations should be considered when interpreting the findings and restrict their generalizability to broader populations. In the hospital cohort, classification was based on retrospective review of radiology and operative reports rather than independent reassessment of the original imaging studies; therefore, interobserver agreement for imaging-based classification could not be evaluated.

Time intervals from hospital admission to diagnosis and, where applicable, from diagnosis or admission to death were not consistently documented across the available records and therefore could not be analyzed.

A major limitation of this study was the restricted clinical and pathological variable set. Information on cardiovascular risk factors, smoking, hypertension, known aortic aneurysm, heritable connective-tissue disorders, bicuspid aortic valve, family history, previous aortic surgery, pregnancy or postpartum status, presenting symptoms, and the interval from symptom onset to hospital presentation was not consistently available. Detailed anatomical and outcome variables, including the location of the primary entry tear, rupture-related complications, hemopericardium, hemothorax, coronary or branch-vessel involvement, malperfusion, treatment course, and cause-specific outcomes, were also not systematically recorded in a comparable manner across both cohorts. Consequently, these variables could not be included in the comparative analysis, and the findings should be interpreted as a comparison limited to demographic characteristics and anatomical classification distributions.

## 5. Conclusions

This study demonstrated significant differences in the distributions of Stanford and DeBakey classifications between hospital-diagnosed cases and cases diagnosed at autopsy. Stanford type A and DeBakey types I–II predominated among cases diagnosed at autopsy, whereas Stanford type B and DeBakey type III were more frequently observed among hospital-diagnosed cases. These findings highlight the value of standardized anatomical classification in both clinical diagnosis and forensic autopsy evaluation.

## Figures and Tables

**Table 1:** Relationship between sex and Stanford/DeBakey classification.

	Male	Female	Total	*p*-Value
Stanford type A	34	12	46	*p* = 0.307
Stanford type B	7	5	12	
Total	41	17	58	
DeBakey type I	14	5	19	*p* = 0.609
DeBakey type II	20	7	27	
DeBakey type III	7	5	12	
Total	41	17	58	

Note: Stanford type A comprises DeBakey types I and II, whereas Stanford type B corresponds to DeBakey type III. *p*-values were calculated using Fisher’s exact test for Stanford classification and the Fisher–Freeman–Halton exact test for DeBakey classification. All *p*-values are two-sided.

**Table 2:** Distribution of Stanford and DeBakey classifications according to diagnostic setting.

	Hospital-Diagnosed Cases	Cases Diagnosed at Autopsy	Total	*p*-Value
Stanford type A	21	25	46	*p* = 0.025
Stanford type B	10	2	12	
Total	31	27	58	
DeBakey type I	10	9	19	*p* = 0.044
DeBakey type II	11	16	27	
DeBakey type III	10	2	12	
Total	31	27	58	

Note: *p*-values were calculated using Fisher’s exact test for Stanford classification and the Fisher–Freeman–Halton exact test for DeBakey classification. All *p*-values are two-sided.

## Data Availability

The datasets generated during and/or analyzed during the current study are available from the corresponding author upon reasonable request.

## References

[1] Basso C., Aguilera B., Banner J., Cohle S., d’Amati G., de Gouveia R.H., di Gioia C., Fabre A., Gallagher P.J., Leone O. (2017). Guidelines for autopsy investigation of sudden cardiac death: 2017 update from the Association for European Cardiovascular Pathology. Virchows Arch..

[2] Mendis S., Puska P., Norrving B. (2011). Global Atlas on Cardiovascular Disease Prevention and Control.

[3] Stecker E.C., Reinier K., Marijon E., Narayanan K., Teodorescu C., Uy-Evanado A., Gunson K., Jui J., Chugh S.S. (2014). Public health burden of sudden cardiac death in the United States. Circ. Arrhythm. Electrophysiol..

[4] Turkish Statistical Institute (2019). Death and Cause of Death Statistics. https://data.tuik.gov.tr/Bulten/Index?p=Olum-ve-Olum-Nedeni-Istatistikleri-2019-33710.

[5] di Gioia C.R.T., Ascione A., Carletti R., Giordano C. (2023). Thoracic Aorta: Anatomy and Pathology. Diagnostics.

[6] Ramanath V.S., Oh J.K., Sundt T.M., Eagle K.A. (2009). Acute aortic syndromes and thoracic aortic aneurysm. Mayo Clin. Proc..

[7] Bossone E., LaBounty T.M., Eagle K.A. (2018). Acute aortic syndromes: Diagnosis and management, an update. Eur. Heart J..

[8] Levy D., Goyal A., Grigorova Y., Farci F., Le J.K. (2022). Aortic dissection. StatPearls.

[9] Olsson C., Thelin S., Ståhle E., Ekbom A., Granath F. (2006). Thoracic aortic aneurysm and dissection: Increasing prevalence and improved outcomes reported in a nationwide population-based study of more than 14,000 cases from 1987 to 2002. Circulation.

[10] Daily P.O., Trueblood H.W., Stinson E.B., Wuerflein R.D., Shumway N.E. (1970). Management of acute aortic dissections. Ann. Thorac. Surg..

[11] DeBakey M.E., Henly W.S., Cooley D.A., Morris G.C., Crawford E.S., Beall A.C. (1965). Surgical management of dissecting aneurysms of the aorta. J. Thorac. Cardiovasc. Surg..

[12] Ding W., Liu Y., Su Z., Li Q., Wang J., Gao Y. (2022). Emerging role of non-coding RNAs in aortic dissection. Biomolecules.

[13] Hagan P.G., Nienaber C.A., Isselbacher E.M., Bruckman D., Karavite D.J., Russman P.L., Evangelista A., Fattori R., Suzuki T., Oh J.K. (2000). The International Registry of Acute Aortic Dissection (IRAD): New insights into an old disease. JAMA.

[14] Evangelista A., Isselbacher E.M., Bossone E., Gleason T.G., Di Eusanio M., Sechtem U., Ehrlich M.P., Trimarchi S., Braverman A.C., Myrmel T. (2018). Insights from the International Registry of Acute Aortic Dissection: A 20-year experience of collaborative clinical research. Circulation.

[15] Isselbacher E.M., Preventza O., Hamilton Black J., Augoustides J.G., Beck A.W., Bolen M.A., Braverman A.C., Bray B.E., Brown-Zimmerman M.M., Chen E.P. (2022). 2022 ACC/AHA guideline for the diagnosis and management of aortic disease: A report of the American Heart Association/American College of Cardiology Joint Committee on Clinical Practice Guidelines. J. Am. Coll. Cardiol..

[16] Howard D.P.J., Banerjee A., Fairhead J.F., Perkins J., Silver L.E., Rothwell P.M., Oxford Vascular Study (2013). Population-based study of incidence and outcome of acute aortic dissection and premorbid risk factor control: 10-year results from the Oxford Vascular Study. Circulation.

[17] Nienaber C.A., Clough R.E. (2015). Management of acute aortic dissection. Lancet.

[18] Yilmaz M., Ozkul B.B., Erdil N., Celbis O. (2026). Acute type A aortic dissection: Assessment of surgically treated and autopsied cases. BMC Cardiovasc. Disord..

[19] Buğra A., Daş T., Buğra A.K., Arslan M.N. (2022). Complications in deaths due to non-traumatic aortic dissection. Bull. Leg. Med..

[20] Palaniappan A., Sellke F. (2022). Medical malpractice litigations involving aortic dissection. J. Thorac. Cardiovasc. Surg..

[21] Melvinsdottir I.H., Lund S.H., Agnarsson B.A., Sigvaldason K., Gudbjartsson T., Geirsson A. (2016). The incidence and mortality of acute thoracic aortic dissection: Results from a whole nation study. Eur. J. Cardiothorac. Surg..

